# Cumulative Payments Through the Earned Income Tax Credit Program in Childhood and Criminal Conviction During Adolescence in the US

**DOI:** 10.1001/jamanetworkopen.2022.42864

**Published:** 2022-11-18

**Authors:** Caitlin A. Moe, Nicole L. Kovski, Kimberly Dalve, Christine Leibbrand, Stephen J. Mooney, Heather D. Hill, Ali Rowhani-Rahbar

**Affiliations:** 1 Department of Epidemiology, University of Washington, Seattle; 2 Daniel J. Evans School of Public Policy & Governance, University of Washington, Seattle; 3 Department of Sociology, University of Washington, Seattle; 4 Department of Pediatrics, School of Medicine, University of Washington, Seattle

## Abstract

**Importance:**

Childhood poverty is associated with poor health and behavioral outcomes. The Earned Income Tax Credit (EITC), first implemented in 1975, is the largest cash transfer program for working families with low income in the US.

**Objective:**

To assess whether cumulative EITC payments received during childhood are associated with the risk of criminal conviction during adolescence.

**Design, Setting, and Participants:**

In this cohort study, the analytic sample consisted of US children enrolled in the 1979 National Longitudinal Study of Youth. The children were born between 1979 and 1998 and were interviewed as adolescents (age 15-19 years) between 1994 and 2016. Data analyses were performed from May 2021 to September 2022.

**Exposure:**

Cumulative simulated EITC received by the individual’s family from birth through age 14 years.

**Main Outcomes and Measures:**

The main outcome was dichotomous, self-reported conviction for a crime during adolescence (age 14-18 years). A cumulative, simulated measure of mean EITC benefits received by a child’s family from birth through age 14 years was derived from federal, state, and family-size differences in EITC eligibility and payments during the study period to capture EITC benefit variation due to differences in policy parameters but not endogenous factors such as changes in household income. Logistic regression models with fixed effects for state and year and robust SEs clustered by mother estimated relative risk of adolescent conviction. Models were adjusted for state-, mother-, and child-level covariates.

**Results:**

The analytical sample consisted of 5492 adolescents born between 1979 and 1998; 2762 (50.3%) were male, 1648 (30.0%) were Black, 1125 (20.5%) were Hispanic, and 2719 (49.5%) were not Black or Hispanic. Each additional $1000 of EITC received during childhood was associated with an 11% lower risk of self-reported criminal conviction during adolescence (adjusted odds ratio, 0.89; 95% CI, 0.84-0.95). Adjusted risk differences were larger among boys (−14.2 self-reported convictions per 1000 population [95% CI, −22.0 to −6.3 per 1000 population]) than among girls (−6.2 per 1000 population [95% CI, −10.7 to −1.6 per 1000 population]).

**Conclusions and Relevance:**

The findings suggest that income support from the EITC may be associated with reduced youth involvement with the criminal justice system in the US. Cost-benefit analyses of the EITC should consider these longer-term and indirect outcomes.

## Key Points

**Question:**

Is family income support received during childhood associated with the risk of criminal conviction during adolescence in the US?

**Findings:**

In this cohort study of 5492 US adolescents, each additional $1000 of cumulative Earned Income Tax Credit received by a child’s family during childhood (age 0-14 years) was associated with 11% lower risk of self-reported criminal conviction during adolescence.

**Meaning:**

The findings suggest that income support for low- and middle-earning families may be associated with reduced risk of adolescent involvement with the criminal justice system.

## Teaser

This cohort study examines the association between cumulative Earned Income Tax Credit payments to families of children aged 0 to 14 years and risk of criminal conviction during adolescence.

## Introduction

An estimated 1 in 6 US children live in poverty (defined as annual income <$25 701 in 2018 for a family of 4).^1^ Family poverty during childhood is associated with adverse behavioral outcomes, including delinquency,^2,3^ which in turn are associated with poorer health and economic outcomes in adulthood.^4^ The association between family income and child and youth behavior operates indirectly through parental stress and investments in safe and well-resourced neighborhoods and schools.^5,6^ Some conceptual models focus on the role of family and environmental stress to explain how parental stress and economic pressure felt by the family are negatively associated with child development and positively associated with adolescent involvement with the juvenile justice system.^7,8^ Other models highlight the resources a family has available to invest in a child, such as in education or housing in safe neighborhoods.^9^

The Earned Income Tax Credit (EITC) is the largest cash transfer program for working families with low income with children in the US.^10^ The federal EITC was first implemented in 1975; as of 2021, 29 states and the District of Columbia have implemented their own supplemental EITC, usually set to a percentage of the federal EITC.^11^ The EITC is available to tax filers with annual earnings below approximately $40 000 and is delivered in a lump sum annual payment at tax time. Approximately 40% of US families with children receive an EITC credit, and in the 2017 tax year, the average EITC payment was $3191 for a family with children.^12,13^ The EITC has been shown in the short term to be associated with reduced poverty,^14^ improved maternal and infant health,^15,16^ and reduced rates of child maltreatment.^17^ Childhood exposure to EITC is associated with improved overall health and educational and employment outcomes in early adulthood.^18,19^

Cumulative EITC exposure during childhood could affect adolescent criminal offending by reducing family economic hardship, which is associated with parental stress, relationship conflict, and poor child behavioral outcomes.^20,21^ In addition, supplemented income may enable a family to modify environmental (ie, neighborhood or school) risk factors, which may, in turn, reduce children’s participation in delinquent activities.^7,22,23^ Multiple pathways between family income support and child development outcomes likely coexist and reinforce each other. Racial and ethnic identity might moderate the association of EITC with the risk of youth involvement in the criminal justice system. Due to strongly rooted racist policies and practices, racial and ethnic minority youths are overrepresented at every level of juvenile justice processing in the US.^24,25^ Sex, race, and ethnicity affect juvenile justice decision-making both jointly and independently.^24,26,27^ Family disadvantage is also known to be disproportionately associated with adverse behavioral outcomes among boys compared with girls, indicating that boys may benefit more from income supplementation.^28^

Using restricted data from the 1979 National Longitudinal Survey of Youth (NLSY79), the Child and Young Adult NLSY, and variation in federal and state EITC policies over 39 years, this study examined the association of the estimated cumulative EITC amount received by a child’s family from birth through age 14 years with that child’s subsequent risk of criminal conviction during adolescence. The design of this investigation relied on exogenous policy changes to simulate a cumulative EITC measurement.

## Methods

### Study Population

The primary data used in this cohort study were from the Child and Young Adult supplements to the NLSY79. The NLSY79 surveyed 12 686 youths aged 14 to 21 years at baseline in 1979.^29^ This original NLSY79 cohort completed follow-up surveys annually until 1994 and then biannually from 1996 through 2016. Participants were surveyed about sociodemographic characteristics, health, attitudes, and behaviors.^30^ Data were analyzed from May 2021 to September 2022. This study was approved by the University of Washington’s Internal Review Board; participants were sent advance letters with information about the study and their participation and gave verbal consent at the beginning of the interview. The study followed the Strengthening the Reporting of Observational Studies in Epidemiology (STROBE) reporting guideline.

The children of mothers who participated in the NLSY79 constitute the NLSY79 Child and Young Adult supplement (CNLSY). As of 2018, at least 10 000 children (ie, index children) had been identified as born to NLSY79 participants and were interviewed as part of the CNLSY in at least 1 survey round.^31^ Members of the CNLSY cohort were born between 1970 and 2014 and were surveyed as children beginning in 1986. Beginning in the 1994 survey year, index children who had reached the age of 15 years or older participated in interviews via self-report as part of the CNLSY Young Adult Survey.

### Measures

#### Criminal Conviction

The criminal conviction outcome was measured by a youth respondent’s positive endorsement in a survey round between the ages of 15 and 19 years to having been convicted of a crime other than a minor traffic violation in the past year. This question was included in the young adult interviews beginning in 1994. Self-reported youth offending has been found to be a reliable indicator of criminal activity and correlates with official records.^8,32^

#### Simulated Cumulative EITC

The federal EITC was first introduced in 1975 and has been expanded several times since. The first state to implement an EITC program was Rhode Island in 1986. Since then, 28 more states have introduced or expanded state EITC programs. Following a method similar to that of Bastian and Michelmore,^18^ we used variation in federal and state EITC credits between 1979 and 2016 to create a cumulative measure of simulated EITC exposure as the key independent variable of interest. Simulated measures of program eligibility or generosity are a common approach to simplifying variation in complex policy rules while isolating variation in program receipt that is not due to individual behaviors or circumstances.^18,33,34,35^ Compared with realized exposure, analyzing simulated exposure offers the advantage of capturing EITC benefit variation due to differences in policy parameters across state, time, and family size but not variation due to endogenous factors such as changes in employment and earnings that are determinants of benefit size and are associated with youth delinquency or conviction.^18^

To construct the simulated measure of mean EITC benefits, we used the National Bureau of Economic Research’s TAXSIM program to calculate the federal EITC for each index child’s mother based on state of residence, marital status, and estimated number of children in the household for each year.^36^ For mothers living in states with EITC policies, we added state EITC payments expressed as a percentage of the federal credit to the total simulated payment. The few state EITCs that are nonrefundable (those that decrease tax liability but are not paid as credits) were not considered EITCs in our analysis because they do not offer direct income support to families at the bottom of the income distribution.^37^ We then drew a nationally representative sample of mothers aged 19 to 45 years from the 1978 Current Population Survey Annual Social and Economic Supplement^38^ and inflated their earnings (including their spouse’s earnings, if married) using the Consumer Price Index for each year during the study period. The earnings distribution was held constant, updating for inflation, to ensure the simulated EITC did not capture year-to-year changes in the earnings distribution that may have been associated with trends in youth conviction rates. Next, we calculated the mean of these EITC benefits for family size (1, 2, or ≥3 children), marital status (married or single), state of residence, and year and assigned each household in the NLSY sample the benefit matching their household characteristics, state, and year. Finally, for each child, we computed the cumulative sum of simulated EITC credits received by that child’s family from age 0 through 14 years. Because the NLSY was administered every other year since 1994, EITC credits for noninterview years were estimated through linear interpolation.

#### Covariates

Individual-level, time-invariant covariates included the child’s sex, race and ethnicity, and a categorical indicator for the child’s year of birth. The primary CNLSY race and ethnicity variable was limited to only 3 categories and was assigned by the NLSY based on the mother’s self-reported race and ethnicity as collected in baseline sampling procedures for NLSY79.^30^ These race and ethnicity categories were coded in the NLSY data as Black, Hispanic, and not Black or Hispanic. Of note, racial and ethnic categories are not biological or fixed but instead are the result of sociopolitical processes and self-identification. Historic and ongoing structural racism in the US economy and criminal justice system make them meaningful categories for this analysis.^39^

The child’s birth cohort was categorical, measured in 5-year bands. The mean number of children in the household was calculated by taking the mean number of children reported in the mother’s household during the waves when the index child was aged 0 to 14 years. Similarly, we used the mothers’ self-reported marital status to create a dichotomized measure of whether the mother was ever married during that age range.

Time-variant state-level variables included in the adjusted models were state gross domestic product, state minimum wage, state unemployment rate, percentage of female-headed households, state prison incarceration rate, and state maximum Temporary Assistance for Needy Families benefit. All state-level variables were measured at the time the index child was age 13 or 14 years. All monetary variables were adjusted for inflation to 2016 US dollars.

### Statistical Analysis

We used logistic regression models for our main analysis with state fixed effects to estimate the association between simulated cumulative EITC exposure and risk of adolescent criminal conviction. Our model was specified with EITC measured as the cumulative simulated EITC benefits that a child’s family received from birth to age 14 years.^18^ Models were adjusted for child-, mother-, and state-level covariates as described. Due to the biannual nature of the NLSY survey, we captured child-level covariates and state of residence from the survey year during which the index child was either 13 or 14 years of age. We applied robust SEs, and due to the substantial number of siblings in the sample, SEs were clustered by the index child’s mother. Risk differences were calculated by computing the mean marginal effect of each additional $1000 in cumulative EITC benefits. All covariates were selected a priori; an alternative model specification is described in the eMethods in the Supplement.

We conducted subgroup analyses to explore whether the association of EITC with adolescent conviction was modified by race and ethnicity. We included an interaction term between EITC exposure (cumulative, ages 0-14 years) and race and ethnicity to assess whether the association of cumulative EITC with the risk of youth conviction was moderated by the mother’s self-reported race and ethnicity. We similarly constructed models to evaluate moderation of the association of cumulative EITC with the risk of adolescent conviction by the child’s sex. The model specification parameters were otherwise identical to the main analysis. As exploratory analyses, we examined whether cumulative EITC was associated with the more serious outcome of conviction for assault charges and with the following outcomes associated with youth delinquency: whether, in the past 12 months, the respondent (1) fought at school or work, (2) took something worth $50 or more that was not theirs, or (3) hit or seriously threatened to hit someone. These questions were not included in survey years after 2012 but were included as an exploratory analysis.

We also conducted robustness checks. We adjusted state-level covariates to correspond to the first year of the child’s life, rather than age 13 or 14 years, and we also explored whether there was moderation of associations by whether the child’s family moved interstate during childhood. All analyses were conducted using Stata, version 15.1 (StataCorp LLC). Two-sided *P* < .05 was considered significant.

## Results

A total of 7930 index children were interviewed at least once between ages 14 and 19 years in the young adult arm of the CNLSY, and 6431 (81.1%) responded to the conviction question in at least 2 survey rounds between ages 14 and 19 years (of 3 possible survey rounds in that period). Of these, 939 (14.6%) were excluded because mothers’ data needed to estimate EITC exposure were missing from 2 or more consecutive survey rounds when the index child was aged 0 to 14 years. The analytic data set consisted of 5492 index children born to 2764 mothers between 1979 and 1998 and surveyed as adolescents from 1994 to 2016; 2730 (49.7%) were female and 2762 (50.3%) were male. As classified by original data collection, 1648 (30.0%) were Black, 1125 (20.5%) were Hispanic, and 2719 (49.5%) were not Black or Hispanic. The mean simulated amount of EITC received by each child’s household between ages 0 and 14 years in 2016 dollars was $10 550 (SD, $5008; range, $697-$28 394) (Table 1).

**Table 1.** Description of the Study Sample

Characteristic	Participants (N = 5492)^a^
**Children**	
Sex	
Female	2730 (49.7)
Male	2762 (50.3)
Race and ethnicity	
Black	1648 (30.0)
Hispanic	1125 (20.5)
Not Black or Hispanic	2719 (49.5)
Year of birth	
1979-1983	1467 (26.7)
1984-1988	1722 (31.4)
1989-1993	1497 (27.3)
1994-1998	806 (14.7)
Convicted during ages 14-18 y	589 (10.7)
Convicted for assault during ages 14-18 y	259 (4.7)
Cumulative EITC during ages 0-14 y, mean (SD), $	10 550 (5008)
**Mothers of children aged 0-14 y**	
Ever married	4556 (83.0)
Children, mean (SD), No.	2.6 (1.2)
Educational level	
High school diploma or lower	3531 (64.3)
Some college or more	1960 (35.7)
Moved to another state	1143 (20.8)
**State characteristics when children were age 13 or 14 y**	
GDP, mean (SD), $, thousands^b^	620.1 (546.0)
Minimum wage, mean (SD), $^b^	7.05 (1.01)
Unemployment rate, mean (SD), %	5.7 (1.7)
Maximum TANF benefit, mean (SD), $, thousands^b^	326.7 (138.2)
Female-headed households, mean (SD), %	36.3 (3.5)
Prison incarceration rate, mean (SD)^c^	427 (136)

Abbreviations: EITC, Earned Income Tax Credit; GDP, gross domestic product; TANF, Temporary Assistance for Needy Families.

^a^ Data are presented as the number (percentage) of participants unless otherwise indicated.

^b^ 2016 US dollars.

^c^ Per 100 000 population.

Overall, each additional $1000 of simulated EITC received during childhood was associated with 11% lower risk of self-reported criminal conviction during adolescence (adjusted odds ratio [OR], 0.89; 95% CI, 0.84-0.95) (Table 2). This estimate translates to a change in the number of adolescent convictions of –10.2 (95% CI, –16.2 to –4.2) per 1000 people for each additional $1000 in cumulative EITC received during childhood.

**Table 2.** Odds Ratios and Risk Differences in Probability of Youth Conviction Associated With Each Additional $1000 of Cumulative EITC Exposure Among 5485 Youths

	Model			
	Crude		Adjusted^a^	
	OR (95% CI)	RD, per 1000 people (95% CI)	OR (95% CI)	RD, per 1000 people (95% CI)
Overall	0.92 (0.90 to 0.94)	−7.8 (−9.6 to −6.0)	0.89 (0.84 to 0.95)	−10.2 (−16.2 to −4.2)
Subgroup analyses				
Sex^b^				
Female	0.93 (0.90 to 0.96)	−4.3 (−6.4 to −2.2)	0.91 (0.84 to 0.97)	−6.2 (−10.7 to −1.6)
Male	0.91 (0.89 to 0.93)	−11.6 (−14.4 to −8.7	0.88 (0.83 to 0.95)	−14.2 (−22.0 to −6.3)
Race and ethnicity^c^				
Black	0.94 (0.90 to 0.97)	−5.5 (−8.4 to −2.6)	0.90 (0.84 to 0.96)	−7.9 (−13.8 to −1.9)
Hispanic	0.92 (0.89 to 0.96)	−8.9 (−13.3 to −4.5)	0.88 (0.81 to 0.96)	−12.5 (−20.9 to −4.1)
Not Black or Hispanic	0.91 (0.88 to 0.93)	−9.0 (−11.7 to −6.2)	0.89 (0.83 to 0.96)	−10.6 (−16.9 to −4.3)

Abbreviations: EITC, Earned Income Tax Credit; OR, odds ratio; RD, risk difference.

^a^ Adjusted for child’s sex, race and ethnicity, and birth cohort; mother’s mean number of children (index child aged 0-14 years) and marital status; and state variables measured when the child was aged 13 to 14 years: state indicator, gross domestic product, prison incarceration rate, minimum wage, unemployment rate, maximum Temporary Assistance for Needy Families benefit, and percentage of households headed by women.

^b^ Omnibus *P* values for group differences: crude model, *P* < .001; adjusted model, *P* < .001.

^c^ Omnibus *P* values for group differences: crude model, *P* = .10; adjusted model, *P* = .14.

We also evaluated whether the association of simulated childhood EITC exposure with risk of self-reported conviction in adolescence was different by sex or by race and ethnicity. As shown in Table 2, the ORs among individual subgroups were similar to the overall OR, although the risk difference for boys was greater than that for girls. Each $1000 in cumulative EITC was associated with a difference of –14.2 (95% CI, −22.0 to −6.3) self-reported convictions per 1000 population among boys and –6.2 (95% CI, −10.7 to −1.6) per 1000 population among girls. Associations were not statistically significantly different when comparing race and ethnicity groups. Similarly, EITC was associated with reduced risk of fighting at school and of hitting or seriously threatening to hit someone (Table 3). There was no association between EITC and stealing something worth more than $50. Our exploratory analysis did not find a significant association between EITC and conviction for assault specifically, but the findings suggested this may merit further inquiry (Table 3). Significant negative associations persisted in analyses with alternate model specifications and robustness checks, presented in eTable 1 and eTable 2 in the Supplement. A correlation matrix for all variables in the adjusted models is shown in eTable 3 in the Supplement. Cumulative EITC was associated with a larger reduction in risk of conviction for adolescents who moved interstate during childhood compared with those who did not move interstate (eTable 1 in the Supplement).

**Table 3.** Odds Ratios and Risk Differences in Probability of Additional Youth Outcomes Associated With Each Additional $1000 of Cumulative Earned Income Tax Credit

Outcome	Model			
	Crude		Adjusted^a^	
	OR (95% CI)	RD, per 1000 people (95% CI)	OR (95% CI)	RD, per 1000 people (95% CI)
Conviction for assault (n = 5485)	0.74 (0.70 to 0.78)	−12.9 (−15.4 to −10.4)	0.86 (0.74 to 1.00)	−5.8 (−11.8 to 0.10)
Fought at school or work (n = 4432)	0.78 (0.76 to 0.80)	−37.1 (−40.7 to −33.6)	0.85 (0.78 to 0.93)	−22.4 (−34.9 to −9.9)
Stole something worth more than $50 (n = 4428)	0.82 (0.78 to 0.86)	−9.2 (−11.7 to −6.8)	0.90 (0.76 to 1.06)	−4.8 (−12.4 to 2.8)
Hit or seriously threatened to hit someone (n = 4429)	0.87 (0.85 to 0.88)	−27.9 (−31.2 to −24.7)	0.92 (0.86 to 0.98)	−16.0 (−28.8 to −3.2)

Abbreviations: OR, odds ratio; RD, risk difference.

^a^ Adjusted for child’s sex, race and ethnicity, and birth cohort; mother’s average number of children (index child aged 0-14 years) and marital status; and state variables measured when child was aged 13 to 14 years: state indicator, gross domestic product, prison incarceration rate, minimum wage, unemployment rate, maximum Temporary Assistance for Needy Families benefit, and percentage of households headed by women.

## Discussion

We evaluated the association between cumulative exposure to EITC credits during childhood and the risk of self-reported criminal conviction during adolescence using intergenerational data from the NLSY79 and CNLSY. By leveraging the longitudinal data and a common method for simulating EITC policy benefits based on marital status, number of dependents, year, and state of residence, we found that each $1000 of simulated cumulative EITC benefits was associated with an 11% decrease in risk of self-reported conviction of a crime during youth.

Previous work evaluating the effects of exposure to EITC found positive associations between the amount of EITC received and reductions in low-weight births,^40^ general child health indicators,^19^ and educational attainment later in life.^18^ Our findings are also consistent with research that measured the longitudinal association of other income supplements received during childhood with youth delinquency. For example, 1 study found that children in households that received casino profit cash transfers were 22% less likely to be arrested for a crime at age 16 or 17 years.^41^

We observed similarly negative associations between EITC and criminal conviction among boys and girls. However, the absolute risk reduction among boys (−14.2 self-reported convictions per 1000 population) was more than twice that among girls (−6.2 per 1000 population). In general, boys are more likely than girls to be involved with the juvenile justice system, and researchers have observed differential treatment throughout juvenile justice processes.^26,42,43^ We did not observe statistically significant differences by race and ethnicity. Exploratory findings of negative associations between EITC and youth fighting are consistent with previous research on EITC and fighting among youths, especially with regard to some variation in outcomes related to school fighting.^44^ In the present study, youth fighting outcomes were included in fewer survey rounds, so these results reinforce our findings of an association between EITC and youth violence but may not be generalizable on their own.

While we did not seek to evaluate the mechanism of effect, other studies on work-based welfare programs have found that positive associations with youth delinquency and behavior problems are primarily mediated through parental stress and parenting practices.^45,46,47^ Families with less economic pressure tend to have lower parental stress and psychological distress, which are in turn associated with warmer and more supportive parenting practices.^48,49^ In addition, supplemented resources enable families to invest in better schooling or housing in better neighborhoods.^9,48^ Policies such as the EITC are important in the effort to narrow health inequities because low- and middle-earning families benefit more than high-earning families from income supplementation. In short, our findings underscore the wide-ranging benefits that family economic support policies can yield for children and youths, which can accumulate into their adult lives.^20,45^

### Strengths and Limitations

This study has strengths, including the large and high-quality longitudinal data set of mothers and their children from 1979 through 2018. Our simulated EITC exposure also captured variation due to differences in policy parameters across state, time, and family size but not due to endogenous factors such as changes in household income.

This study also has limitations. The study population was not a nationally representative cohort of children, so our findings may not be generalizable to all US adolescents. Consistent with best practices for the CNLSY cohort,^50^ our analysis was unweighted. It follows that our results represent the associations present in the CNLSY cohort itself but not in any broader population. Our findings may be subject to selection bias as not every participant in the study was able to be interviewed each year, and those who were lost to follow-up were likely to be systematically different from those who stayed in the study in ways (eg, mobility, incarceration) that were likely to have affected the outcome. Some individuals were incarcerated and could not be interviewed in certain survey years. However, NLSY interviewers were usually able to interview these individuals at least once. Conviction during adolescence was self-reported, and some youths may not have known whether they were convicted of a crime or routed through deterrence programs. Moreover, juvenile justice is an institutional process with results that are shaped by family resources and racial discrimination.^43^

## Conclusions

The findings of this study showed an association between income support for low- and middle-earning families and reduced risk of adolescent involvement with the criminal justice system in the US. Economic policies such as the EITC may be helpful to reduce a multitude of inequities in socioeconomic determinants of health, with effects lasting into adulthood.

## Notes

Supplement.
eMethods.
**eTable 1.** Sensitivity Analyses and Robustness Checks for Relative Risk and Risk Differences in Probability of Youth Conviction for Assault Associated With Each Additional $1000 of Cumulative EITC Exposure **eTable 2.** Full Adjusted Model (A Priori Analysis) Predictors, Overall Conviction **eTable 3.** Pairwise Correlation Matrix
