# Supplementary material for: Cumulative Payments Through the Earned Income Tax Credit Program in Childhood and Criminal Conviction During Adolescence in the US
Source: JAMA Netw Open. 2022 Nov 18;5(11):e2242864. doi: 10.1001/jamanetworkopen.2022.42864 (PMC9675000; doi:10.1001/jamanetworkopen.2022.42864)
Supplement: Supplement. — eMethods. eTable 1. Sensitivity Analyses and Robustness Checks for Relative Risk and Risk Differences in Probability of Youth Conviction for Assault Associated With Each Additional $1000 of Cumulative EITC Exposure eTable 2. Full Adjusted Model (A Priori Analysis) Predictors, Overall Conviction eTable 3. Pairwise Correlation Matrix [file jamanetwopen-e2242864-s001.pdf]

Supplementary Online Content

Moe CA, Kovski NL, Dalve K, et al. Cumulative payments through the Earned Income Tax Credit program in childhood and criminal conviction during adolescence in the US. *JAMA Netw Open*. 2022;5(11):e2242864. doi:10.1001/jamanetworkopen.2022.42864

eMethods.
eTable 1. Sensitivity Analyses and Robustness Checks for Relative Risk and Risk Differences in Probability of Youth Conviction for Assault Associated With Each Additional $1000 of Cumulative EITC Exposure
eTable 2. Full Adjusted Model (A Priori Analysis) Predictors, Overall Conviction
eTable 3. Pairwise Correlation Matrix

This supplementary material has been provided by the authors to give readers additional information about their work.

© 2022 Moe CA et al. *JAMA Network Open*.

eMethods

Our *a priori* specified model was a robust Poisson regression model of the dichotomous risk of conviction for each adolescent in our cohort. However, Poisson regression models assume the mean of the underlying distribution is the same as its variance. A logistic regression model was determined to be a better fit for the data and its estimates, which were very similar, are presented in the main manuscript. Results of sensitivity analyses, performed under the Poisson model specifications, are presented in eTable 1. The full results of the Poisson regression model are presented in eTable 2. A correlation matrix of all variables included in the models is shown in eTable 3.

© 2022 Moe CA et al. *JAMA Network Open*.

eTable 1. Sensitivity Analyses and Robustness Checks: Relative Risk and Risk Differences in Probability of Youth Conviction for Assault Associated With Each Additional $1000 of Cumulative EITC Exposure, N=5485

**Analysis**	**RR**	**95% CI**		**RD**	**95% CI**
State-level covariate adjustments					
State fixed effects from first year	0.90	0.86, 0.95		-11.1	-16.7, -5.4
State covariates, fixed effects from child's first year (age 0-1)	0.93	0.87, 0.99		-7.9	-14.9, -1.0
Subgroups based on interstate movement					
Did not move interstate (age 0-14)	0.92	0.87, 0.98		-8.4	-14.3, -2.5
Moved interstate (age 0-14)	0.89	0.83, 0.95		-14.7	-23.0, -6.3

All models adjusted for: child's sex, race, birth cohort; mothers' average number of kids (index child age 0-14), and marital status; and state variables: state indicator, state gross domestic product, state prison incarceration rate, minimum wage, unemployment rate, maximum TANF benefit, and percentage of households headed by women

CI = Confidence interval; RD = Risk difference, computed per 1,000 people; RR = Relative risk

© 2022 Moe CA et al. *JAMA Network Open*.

eTable 2. Full Adjusted Model (A Priori Analysis) Predictors, Overall Conviction

Variable	RR	95% CI	P-value
EITC, $1000s	0.91	0.86 – 0.96	0.001
Race/ethnicity			
Hispanic	Ref		
Black	0.70	0.53 – 0.92	0.011
Non-Black, non-Hispanic	0.89	0.71 – 1.13	0.337
Average # children	1.19	1.11 – 1.27	<0.001
Mother marital status	0.54	0.44 – 0.66	<0.001
Child sex	0.48	0.41 – 0.56	<0.001
Birth cohort			
1979-1983	Ref		
1984-1988	1.33	0.98 – 1.80	0.068
1989-1993	1.75	1.05 – 2.90	0.030
1994-1999	1.94	0.87 – 4.30	0.104
State prison incarceration rate	1.00	0.996 – 1.00	0.031
State gross product	1.00	0.999 – 1.00	0.821
State minimum wage	0.95	0.83 – 1.09	0.431
State unemployment rate	0.94	0.86 – 1.03	0.212
State maximum TANF benefit	1.00	0.99 – 1.00	0.789
State percent female-headed households	0.93	0.84 – 1.03	0.148
State of residence	[individual state estimates suppressed per data contract]		

CI = Confidence interval; RD = Risk difference, computed per 1,000 people; RR = Relative risk

© 2022 Moe CA et al. *JAMA Network Open*.

eTable 3. Pairwise Correlation Matrix

	Conv.	EITC	Sex	Race	AvgKids	Married	Cohort	PrisonRate	GSP	MinWage	Unempl	TANF	FHH	State
Conviction	1.000													
EITC	-0.118	1.000												
Sex	-0.124	-0.009	1.000											
Race	-0.032	0.091	0.006	1.000										
AvgKids	0.029	0.299	0.007	-0.129	1.000									
Married	-0.066	0.039	0.008	0.166	-0.083	1.000								
Cohort	-0.114	0.922	-0.014	0.086	0.200	0.013	1.000							
PrisonRate	-0.059	0.125	0.005	-0.184	0.025	-0.037	0.180	1.000						
GSP	-0.040	0.162	0.011	-0.283	0.069	0.007	0.172	0.201	1.000					
MinWage	-0.038	0.300	-0.003	0.005	0.095	-0.010	0.334	-0.200	0.294	1.000				
Unempl	-0.045	0.150	0.017	-0.075	0.048	-0.044	0.212	0.051	0.249	0.231	1.000			
TANF	-0.008	0.224	0.006	0.003	0.070	0.031	0.141	-0.409	0.447	0.444	0.112	1.000		
FHH	-0.131	0.622	0.005	0.020	0.130	-0.057	0.622	0.150	0.184	0.272	0.254	0.103	1.000	
State	0.026	0.022	-0.012	0.119	-0.019	0.041	-0.028	-0.059	-0.262	-0.244	-0.140	-0.123	-0.108	1.000

© 2022 Moe CA et al. *JAMA Network Open*.
